# Supplementary material for: Evidence for continuity of interstitial spaces across tissue and organ boundaries in humans
Source: Commun Biol. 2021 Mar 31;4:436. doi: 10.1038/s42003-021-01962-0 (PMC8012658; doi:10.1038/s42003-021-01962-0)
Supplement: Supplementary file 2 — Supplementary Information [file 42003_2021_1962_MOESM2_ESM.pdf]

Supplemental Figures

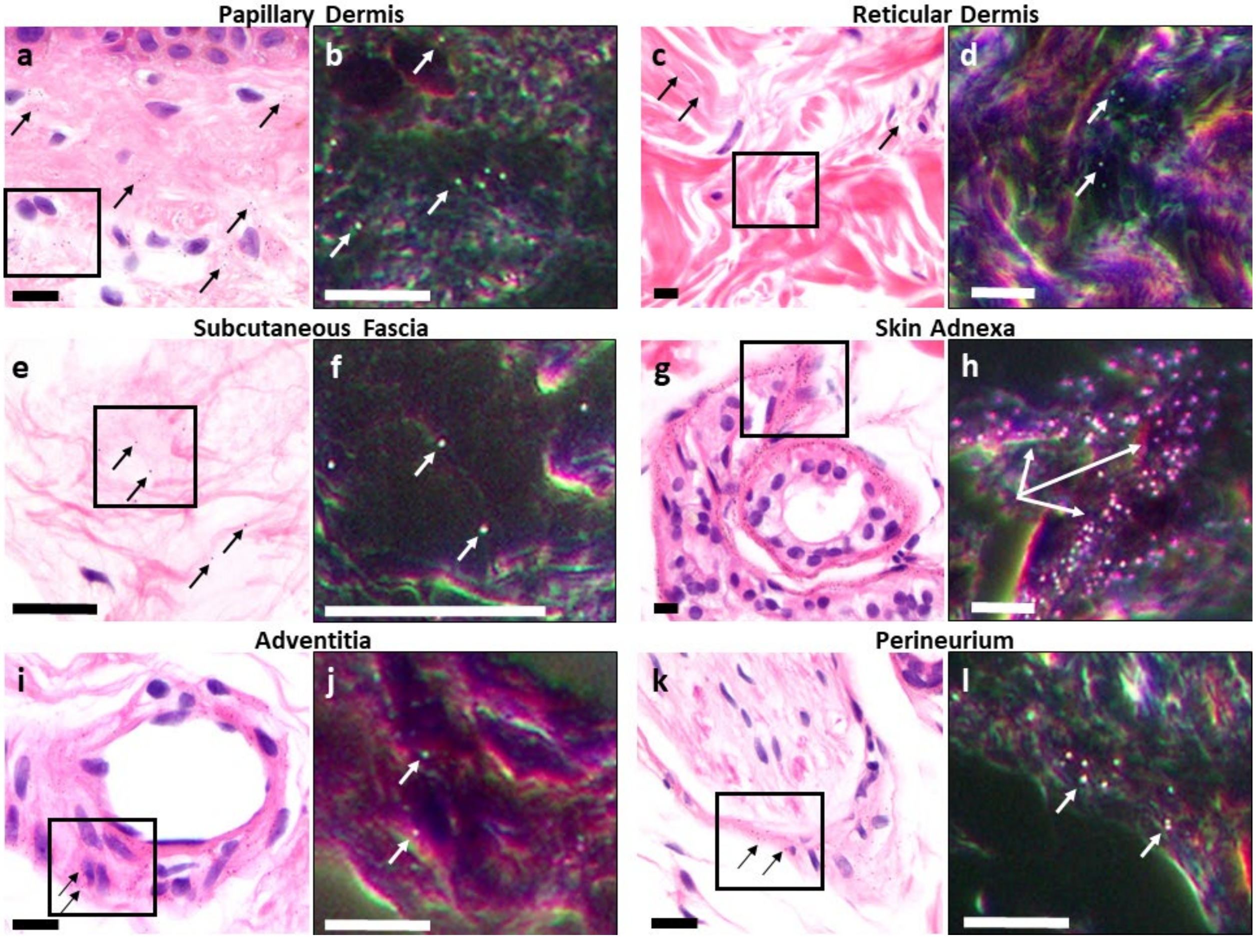

Supplemental Figure 1. Topically-applied colloidal silver particles are found in the subcutaneous fascia. Juxtaposed light field and dark field microscopy of regions of the same H&E sections: epidermis and papillary dermis (**a,b**), reticular dermis (**c,d**), subcutaneous fascia (**e,f**), adnexa (**g,h**), arteriole with adventitia (**i,j**) and peripheral nerve (**k,l**). Silver particles (which appear as very fine brown-black granules on light microscopy, some labeled with black arrows, and bright granules on dark field microscopy, some labeled with white arrows) are identified in the interstitial spaces of papillary and reticular dermis, and those of the subcutaneous fascia, as well as in the basement membrane of adnexal structures, in perivascular adventitia, and in perineurium. Scale bars = 50 μM.

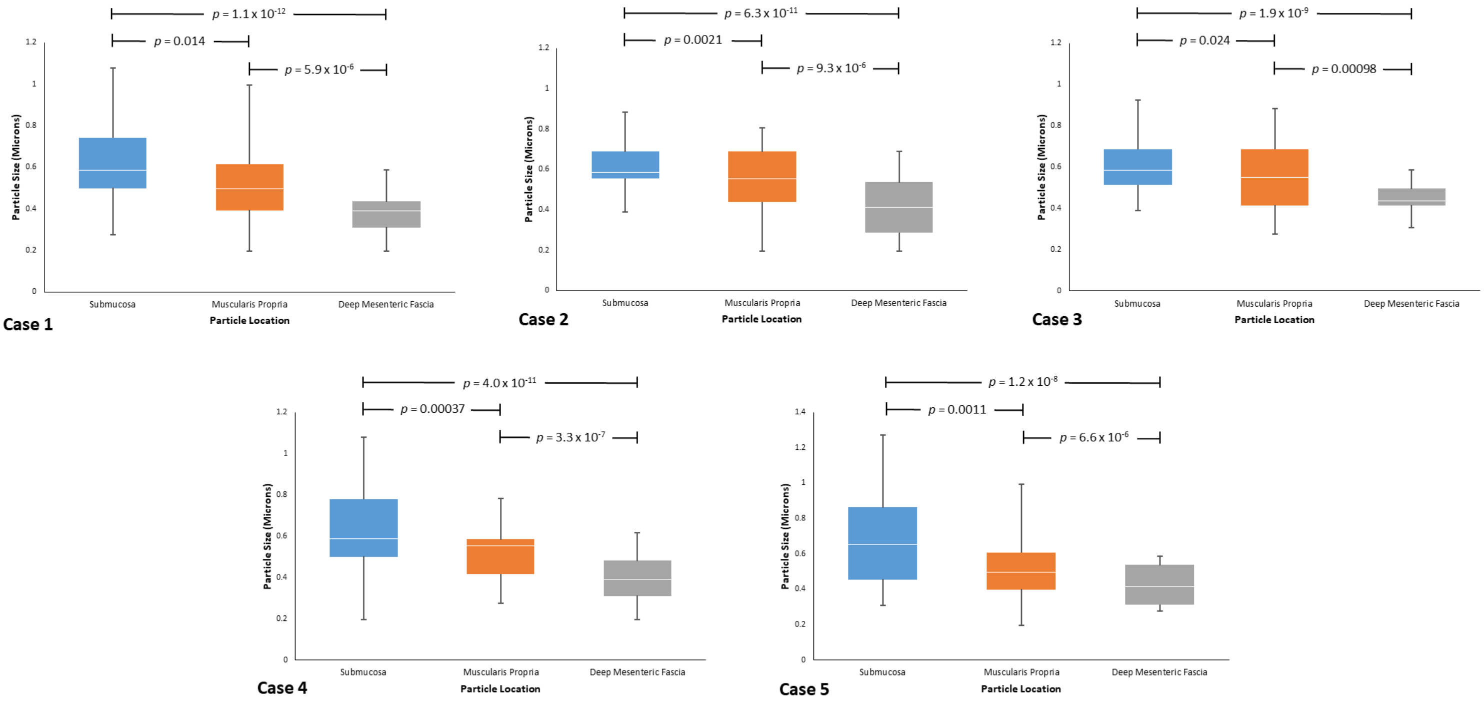

Supplemental Figure 2. Tattoo pigment particle distribution in each colon specimen. Distribution of particles by size in compartments at increasing distance from lumen in aggregated data from each colon tattoo specimen with 50 particles sized for each of the 3 tissue layers per specimen. The boxes represent first, median, and third quartiles; the bars represent the range of data points.

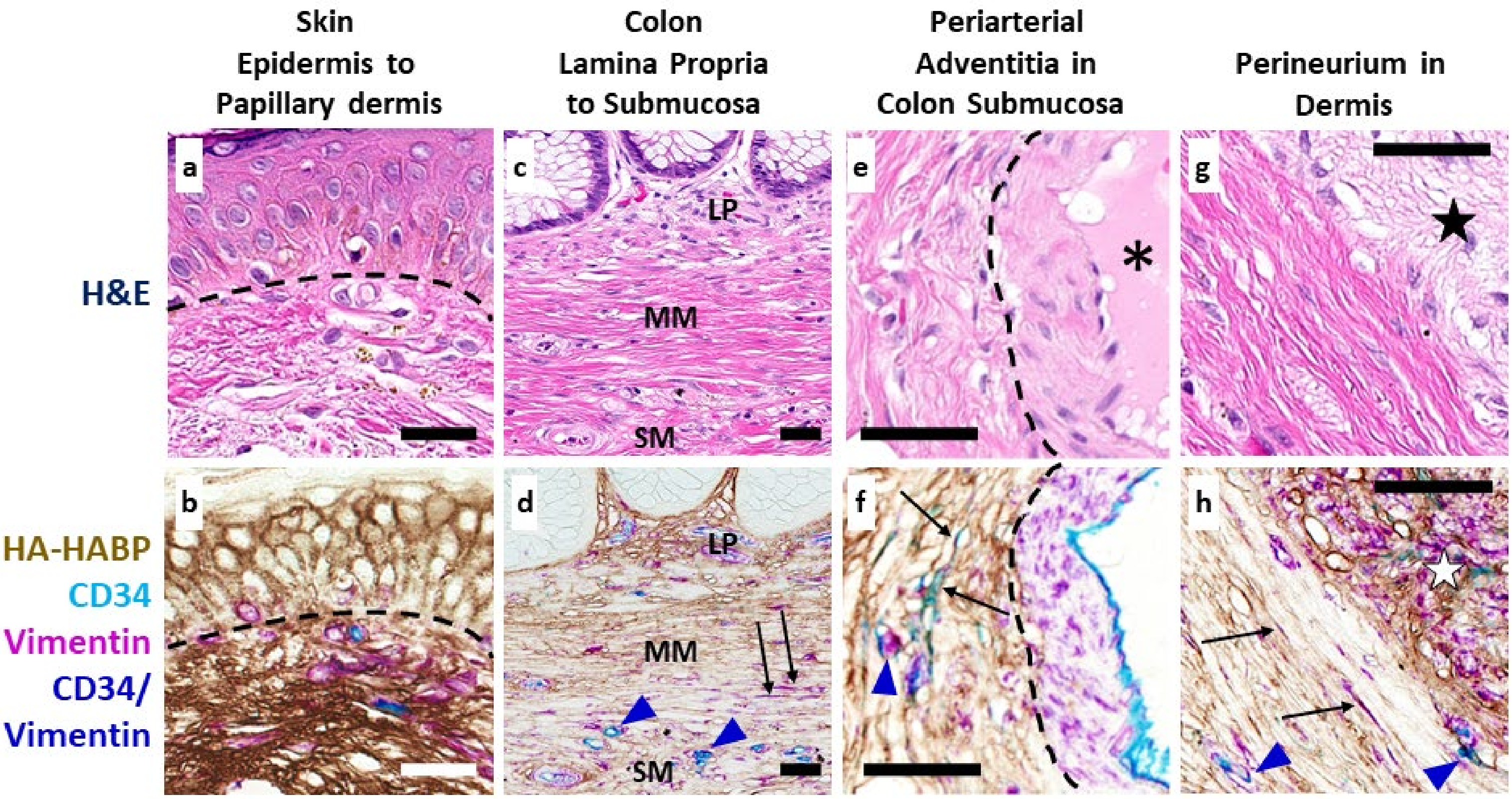

Supplemental Figure 3. Hyaluronic acid is a marker of interstitial spaces. Full thickness skin and colon samples were stained with H&E (top panels), then decolorized and restained using a multiplex chromogenic assay HABP (brown), vimentin (magenta), and CD34 (teal), CD34/Vimentin overlap (navy blue). **a,b** HA in interstitial spaces between epidermal keratinocytes (above dotted line) and between collagen bundles of the papillary dermis (below dotted line). **c,d** HA in interstitial spaces of the lamina propria (LP) and in channels through the muscularis mucosae (MM) and submucosa (SM). **e,f** HA in interstitial spaces of the adventitia around an artery (lower right, demarcated by dotted line) in the wall of the colon. The lumen of the artery is filled with red blood cells (*, upper panel) and the muscular wall

of the artery is highlighted by vimentin staining (magenta; lower panel). **g,h** Interstitial spaces of perineurium and fibroconnective tissue around the nerve (star) in the colonic wall stain for HA, as do the intercellular spaces in the nerve itself. In all multiplex stained images, vimentin staining highlights fibroblasts, mononuclear inflammatory cells, vascular smooth muscle, nerve, vascular endothelial cells, and interstitial lining cells. CD34 stains endothelial cells and interstitial lining cells. Co-localization between CD34 and vimentin (navy blue) indicates either vascular endothelium, when lining capillaries or arteriovenous structures (navy blue arrowheads), or interstitial lining cells when along collagen bundles. Scale bars = 100 μM.

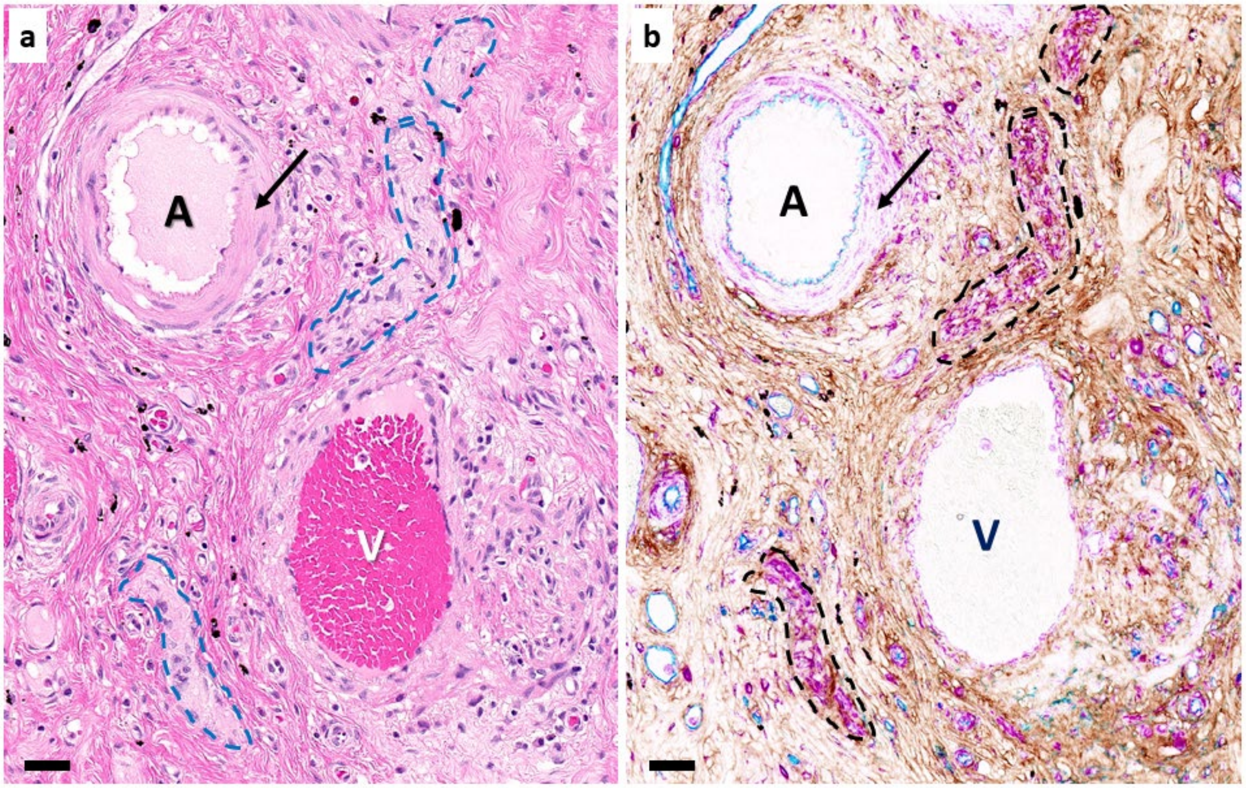

Supplemental Figure 4. Continuity between interstitial spaces, perivascular adventitia and perineurium in colonic submucosa. a H&E section of colonic submucosa with artery (A), vein (V), and peripheral nerve (dashed lines) Arterial tunica media (muscular coat) labeled by arrow. **b** HABP-vimentin-CD34 triplex chromogenic stain of the same tissue section after de-colorization (HABP, brown; vimentin, magenta; CD34, teal). Nerves show strong vimentin staining as does the tunica media (of the artery (arrow). HABP highlights HA in interstitial spaces, showing continuity of spaces between connective tissue of the bowel and the perivascular and perineurial stroma. A, artery; V, vein. In all multiplex-stained images, staining is as in Fig. 5. Scale bars = 100 μM.

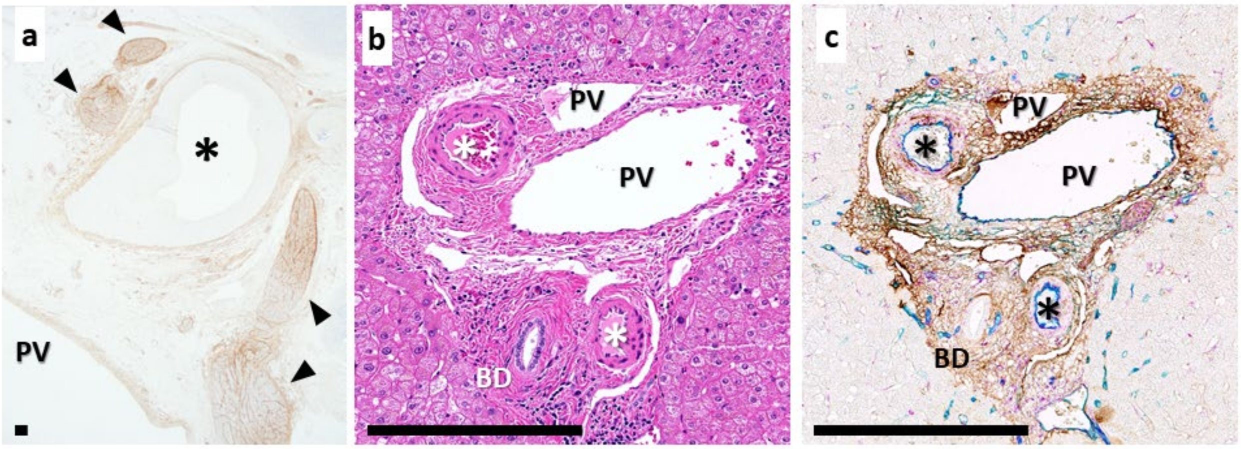

Supplemental Figure 5. Continuity of extrahepatic interstitial spaces of the porta hepatis and intrahepatic spaces within the "space of Mall" demonstrated by HA localization. **a** HA staining of interstitial spaces of structures of the portal hepatis: the adventitia of portal vein (PV), hepatic artery (*) and within and around nerves (arrowheads). (Single stain of HA by HABP binding, DAB). **b** H&E section of liver with portal triad: hepatic artery (*), portal vein (PV), and bile duct (BD). **c** Multiplex chromogenic assay of the same region. HABP (brown), vimentin (magenta), CD34 (teal). HABP staining is coincident with the space of Mall; it highlights HA in interstitial spaces, showing continuity between all compartments of the space of Mall. In all multiplex stained images, staining is as in Fig. 5; interstitial lining cells are in the space of Mall. Scale bars = 400 μM.

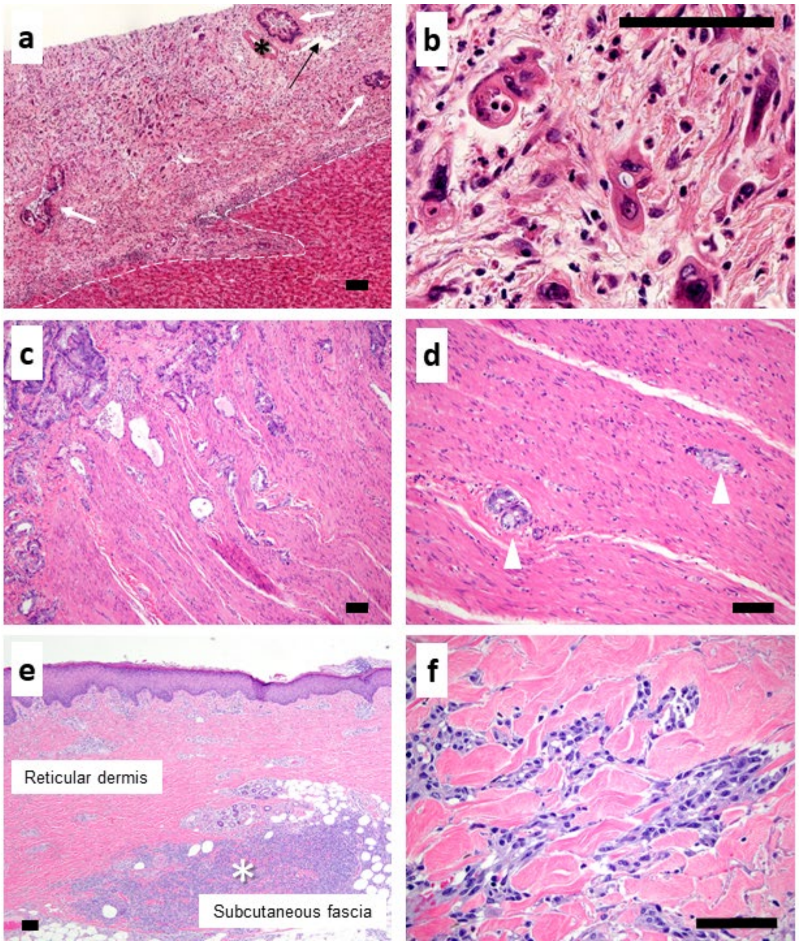

Supplemental Figure 6. Interstitial spaces are a route of spread for malignant tumors. **a** Periductal spread of hilar cholangiocarcinoma through the space of Mall of the portal tract stroma in the liver. Note the absence of invasion through the periportal limiting plate (dotted line) with tumor confined completely within the portal tract stroma, surrounding many portal structures, but not invading them, i.e. bile ducts (white arrows), hepatic artery (*) and portal vein (black arrow). **b** Cholangiocarcinoma within interstitial spaces of portal tract stroma; same tumor as **a**. **c** Colonic adenocarcinoma (dark blue/purple glands percolating from upper left, downward) infiltrating through the submucosa and between muscle bundles of the muscularis propria. **d** Same tumor as **c** showing tumor nests (arrow heads) between pink muscle bundles without desmoplastic reaction. **e** In-transit malignant melanoma tumor deposit (*) in deep reticular dermis and subcutaneous fascia, several centimeters away from the primary lesion. **f** Higher

magnification view of melanoma (grey and blue cells) in **e** infiltrating through interstitial spaces of reticular dermis between its pre-existing, pink, acellular collagen bundles. All images H&E. Scale bars = 100 μM.
