# Supplementary material for: Evidence for continuity of interstitial spaces across tissue and organ boundaries in humans
Source: Commun Biol. 2021 Mar 31;4:436. doi: 10.1038/s42003-021-01962-0 (PMC8012658; doi:10.1038/s42003-021-01962-0)
Supplement: Supplementary file 3 — Description of Additional Supplementary Files [file 42003_2021_1962_MOESM3_ESM.pdf]

Description of Additional Supplementary Files

File Name: Supplemental Data 1

Description: Source data for tattoo pigment particle sizes in colon specimens by region. SM: submucosa, MP: muscularis propria, Deep: deep mesenteric fascia, Std Dev: standard deviation.
